# The Tumor Suppressor p53 Downregulates p107 (*RBL1*) Through p21–RB/E2F Signaling and Tandem E2F Sites

**DOI:** 10.3390/ijms26209903

**Published:** 2025-10-11

**Authors:** Khaled Azzahrani, Faleh Alqahtani

**Affiliations:** 1 Central Laboratory, College of Pharmacy, King Saud University, Riyadh 11451, Saudi Arabia; 2 Department of Pharmacology and Toxicology, College of Pharmacy, King Saud University, Riyadh 11451, Saudi Arabia; afaleh@ksu.edu.sa

**Keywords:** *RBL1*, p107, E2F transcription factors, p53, cell cycle regulation

## Abstract

*RBL1* (p107) is a member of the retinoblastoma (RB) family of pocket proteins involved in cell cycle regulation and E2F transcriptional repression. While its promoter contains conserved E2F motifs, the integrated regulation of *RBL1* by upstream tumor suppressor pathways remains incompletely understood. Here, we investigate the p53-dependent transcriptional regulation of *RBL1* and dissect the contribution of its tandem E2F binding sites to this mechanism. Luciferase assays in synchronized cells demonstrated that these two conserved E2F sites are required for cell cycle-dependent activation of the *RBL1* promoter. Overexpression of p53 showed that p53 represses *RBL1* promoter activity in an E2F site-dependent manner. Using HCT116 p21 knockout cells, we revealed that this p53-dependent repression is mediated by p21. Chromatin immunoprecipitation confirmed dynamic in vivo binding of E2F1–3 and E2F4, while DNA pull-down assays revealed specific in vitro recruitment of RB, p107, and E2F1-4 to the two E2F sites, along with weak binding of MuvB components. Additional experiments in RB^–/–^ and LIN37^–/–^ knockouts showed that RB/E2F repressing complex plays the main role in repressing the *RBL1* promoter, while E2F4, p107, and p130 can support this effect to a lesser extent. Overall, our findings demonstrate that p53 controls *RBL1* expression indirectly through the p21–RB–E2F pathway by utilizing two E2F binding sites within the *RBL1* promoter.

## 1. Introduction

As the central guardian of genomic stability, the tumor suppressor p53 orchestrates diverse cellular responses to stress, such as DNA damage, oncogenic signaling, and hypoxia [1,2]. When triggered, p53 can endorse growth suppression through multiple routes—most notably by inducing cell cycle arrest, initiating programmed cell death, or promoting cellular senescence [3,4,5]. One of the primary mechanisms by which p53 mediates these effects is through the transcriptional activation of downstream effectors, particularly p21 (*CDKN1A*), a potent inhibitor of cyclin-dependent kinases (CDKs) [6]. Acting downstream of p53, p21 halts cell cycle progression by directly inhibiting various cyclin-CDK complexes, thereby imposing a blockade at the G1/S checkpoint [7].

Another major regulator of the eukaryotic cell cycle is the retinoblastoma (RB) protein family, which includes RB (*RB1*), p107 (*RBL1*), and p130 (*RBL2*) [8]. These so-called pocket proteins serve as transcriptional repressors, particularly by binding and regulating E2F transcription factors—a critical interaction that governs the transition from G1 to S phase [9]. In their hypophosphorylated form, RB family proteins form inhibitory complexes with E2Fs, preventing the transcription of genes required for DNA synthesis and cell cycle progression. Upon CDK-mediated phosphorylation in late G1, the pocket proteins are inactivated, releasing E2Fs and allowing transcriptional activation of S-phase genes [10,11,12,13]. Adding to this network is the DREAM complex (DP, RB-like, E2F, and MuvB), which typically forms when p130 or p107 associates with E2F4 and the MuvB core, functioning predominantly during quiescence and early G1 to repress a broad suite of cell cycle genes [13,14,15,16].

The tumor suppressor p53 represses expression of E2F target genes indirectly through the p53–p21–RB/DREAM axis [16,17,18]. In this model, p21 inhibits CDK activity, leading to hypophosphorylation and hence activation of RB family proteins, which then repress transcription through E2F sites. This elegant mechanism helps maintain the integrity of the G1/S checkpoint under stress conditions. However, while this pathway is well-characterized at a high level, the detailed promoter architecture and relative contributions of individual RB family proteins or repressor complexes remain poorly resolved, especially for many E2F-responsive genes.

Interestingly, within the RB protein family, p107 (*RBL1*) displays a set of distinct regulatory features. Unlike RB, whose expression is generally constitutive and primarily modulated through post-translational mechanisms, p107 expression is regulated mainly at the transcriptional level [19,20,21]. Its expression is tightly cell cycle-regulated, with both mRNA and protein levels remaining low during quiescence but rising sharply during late G1 and S phase [21,22]. The p107 promoter contains two highly conserved tandem E2F binding sites, both of which appear to be necessary for proper activation and repression in a cell cycle-dependent manner. Interestingly, in humans, both sites match the E2F consensus sequence exactly, indicating strong potential responsiveness to E2F-based transcriptional regulation [20,23].

Functionally, p107 has been shown to compensate for RB loss in a variety of experimental systems. For instance, double-knockout mice lacking both RB and p107—or RB and p130—develop retinoblastoma, whereas single knockouts do not, indicating that these proteins have overlapping tumor suppressor functions in retinal development [24,25,26,27,28,29,30]. This compensatory effect is not limited to the retina but has also been observed in other tissues, such as cardiac, pulmonary, and epidermal cells [31,32,33]. In fact, the loss of RB leads to transcriptional upregulation and post-transcriptional stabilization of p107 in various cell types, providing evidence for a feedback compensatory mechanism rather than mere functional redundancy [28,34,35,36].

Although p107’s role in tumor suppression is increasingly appreciated, the mechanisms through which p107 itself is transcriptionally repressed in response to p53 activation remain incompletely understood. Recently, genome-wide data have demonstrated that *RBL1* is regulated by both RB and the DREAM complex [37,38]. However, the precise mechanisms underlying this regulation—and in particular, the relative contributions of RB versus DREAM—have not been systematically characterized. Moreover, the specific role of the tandem E2F elements within the *RBL1* promoter in mediating this repression has not yet been addressed.

In this study, we sought to fill this knowledge gap by systematically investigating how p53 regulates *RBL1* transcription. Specifically, we aimed to determine whether *RBL1* expression is repressed by p53 induction, and whether that repression depends on p21, and to examine the specific role of the two tandem E2F binding sites within the *RBL1* promoter in mediating this effect. We also set out to identify the protein complexes—such as RB and components of the DREAM complex—that are recruited to these E2F motifs under different cellular conditions. Finally, we wanted to clarify the relative contributions of RB and DREAM to *RBL1* repression in the context of p53 activation, with the goal of unravelling their individual and potentially cooperative roles in this regulatory pathway.

## 2. Results

### 2.1. p53 Represses RBL1 Expression in a p21-Dependent Manner

The p53 pathway is essential for halting cell cycle progression by inducing p21 (*CDKN1A*), a strong CDK inhibitor, that, in turn, suppresses the transcription of many cell cycle-related genes. This indirect repression is known to occur via the p53–p21–RB/DREAM axis, which reduces the phosphorylation of pocket proteins, leading to repression of E2F target genes [16,18]. Genome-wide data have already suggested that *RBL1* belongs to this regulatory class and listed *RBL1* as a target of p53–p21–RB/DREAM signaling [37,38]. However, direct mechanistic validation in human cancer cell systems has remained limited.

**Figure 1:**
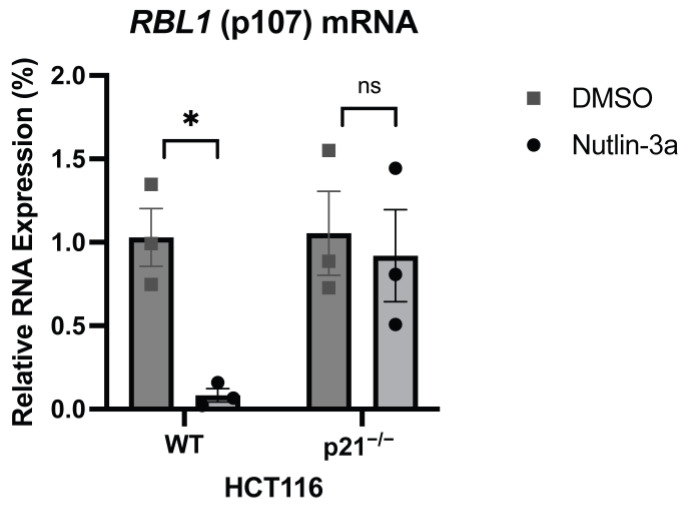
p53 Represses *RBL1* Expression in a p21-Dependent Manner. Real-time RT-qPCR analysis of *RBL1* (p107) mRNA levels in human colon carcinoma HCT116 wild-type (WT) and p21-deficient (p21^−/−^) cells. Cells were treated with Nutlin-3a (a p53 activator) for 48 h or DMSO as a control. Expression levels were normalized to U6 as an internal control, and relative expression was calculated using the ΔΔCt method and normalized to DMSO. Mean values ± SEM from three biological replicates are shown. Significance was tested using Student’s *t*-test (ns: not significant; *: *p* ≤ 0.05). Confirmation of the knockouts are available in Supplementary Figure S1.

To confirm this regulation experimentally, we treated HCT116 colon carcinoma cells, both wild-type (WT) and p21 knockout (p21^−/−^), with Nutlin-3a, a compound that stabilizes and activates p53 by inhibiting MDM2 [39]. In WT cells, Nutlin-3a led to a robust increase in p21 mRNA, as anticipated. Unsurprisingly, this induction was absent in p21^−/−^ cells, confirming the successful genetic knockout (Supplementary Figure S1). Conversely, *RBL1* mRNA levels dropped significantly upon Nutlin-3a treatment in WT cells. However, in the absence of p21, this repression completely disappeared (Figure 1). These results therefore confirm that *RBL1* is repressed by p53 in a strictly p21-dependent manner.

### 2.2. p53-Mediated Regulation of RBL1 Is Retained in a Short Promoter Fragment Containing Conserved E2F Sites

We then explored whether a specific promoter region might account for the p53-driven repression of *RBL1*. For this, we constructed two luciferase reporters: one driven by a 2960 bp upstream fragment of the human *RBL1* promoter and another using only a 400 bp segment proximal to the transcription start site. These constructs were co-transfected into HCT116 p53^−/−^ cells along with either a p53-wt or p53-mut expression plasmids.

**Figure 2:**
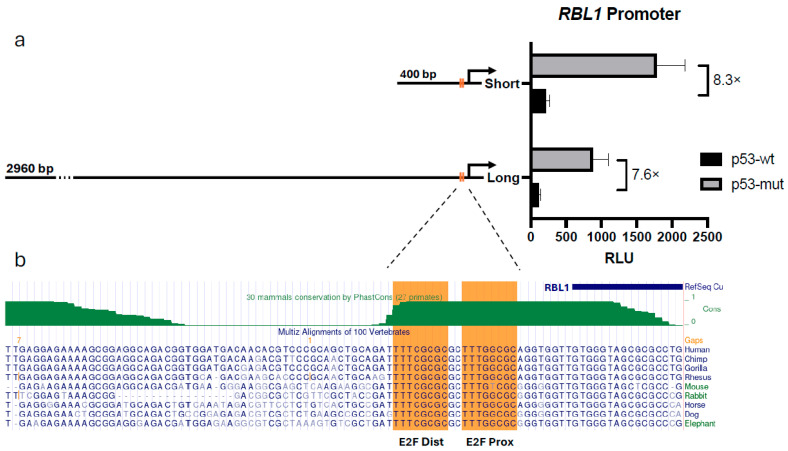
p53-mediated Regulation of *RBL1* Is Retained in a Short Promoter Fragment Containing Conserved E2F Sites. (**a**) Luciferase reporter assay measuring the activity of the human *RBL1* promoter. HCT116 p53^−/−^ cells were transfected with reporter plasmids containing either a long (2960 bp) or a short (400 bp) fragment of the *RBL1* promoter, and co-transfected with either p53-wt or p53-mut expressing plasmids. Relative light units (RLUs) were calculated as the ratio of firefly luciferase activity from the promoter reporter constructs to the Renilla luciferase activity from a co-transfected control plasmid. One representative experiment from two biological replicates is shown, demonstrating that the shorter promoter fragment retains p53-dependent repression. (**b**) UCSC Genome Browser snapshot displaying the location and evolutionary conservation of the two tandem E2F binding sites within the human *RBL1* promoter. This phylogenetic conservation analysis highlights the critical nature of these elements within the minimal promoter region.

Both the full-length and the short promoter fragments showed a similar degree of repression in response to p53 (Figure 2a). This suggested that the 400 bp region was not only sufficient but possibly the main driver of the observed regulatory effect. Based on these findings, we proceeded with the shorter promoter fragment and excluded the longer construct from further analysis.

To examine this region more closely, we turned to the UCSC Genome Browser to assess its evolutionary conservation [40]. It led to identification of two tandem E2F binding motifs which are strongly conserved across vertebrates (Figure 2b). These tandem E2F sites in the *RBL1* promoter have previously been shown to be essential for its regulation by RB family proteins [20,23]. Building on this, we sought to confirm their contribution to p53-mediated repression and to test whether both sites are required to transmit the p53–p21 signal.

### 2.3. E2F Binding Sites Are Essential for Cell Cycle–Dependent Regulation of RBL1

The contribution of the tandem E2F binding sites to *RBL1* regulation has been reported previously, where mutation of these sites disrupted repression in quiescent cells and abolished proper promoter activity during cell cycle progression [20]. To confirm the contribution of these E2F sites in the cell cycle-dependent regulation of *RBL1*, we monitored first how *RBL1* itself is expressed during the cell cycle using synchronized cells. NIH3T3 fibroblasts were serum-starved for 62 h to induce quiescence (G_0_) and then re-stimulated with serum addition. We collected cells every 4 h after stimulation and measured mRNA levels of *RBL1*, *CCNE2* (Cyclin E2, an early G1/S gene), and *CDC25C* (as a G2/M gene). Cell cycle phases were assessed by flow cytometry (FACS) analysis (see Supplementary Figure S2).

**Figure 3:**
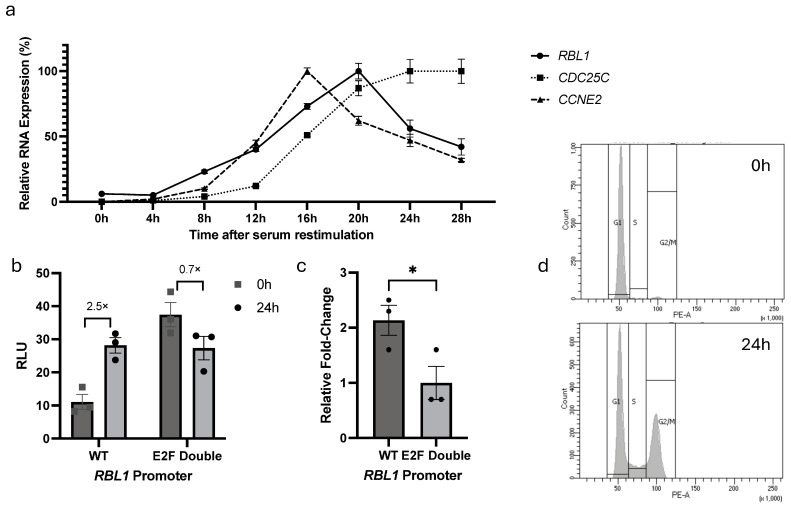
E2F Binding Sites Are Required for Cell Cycle–Dependent Regulation of *RBL1* Expression. (**a**) Expression of *RBL1*, *CCNE2* (Cyclin E2), and *CDC25C* mRNA over the course of one cell cycle. Mouse NIH3T3 cells were arrested in G0 by serum starvation for 62 h. To stimulate cells to re-enter the cell cycle, fetal calf serum (FCS) was added to the medium. Cells were collected every 4 h after serum restimulation. mRNA levels were quantified by real-time RT-qPCR and normalized to U6. Mean values ± SEM from two technical replicates are given. One representative experiment from two biological replicates is shown. Approximate cell cycle phases were assessed by FACS analysis (see Supplementary Figure S2). (**b**) Luciferase reporter assay demonstrating the cell cycle-dependent regulation of *RBL1* promoter. NIH3T3 cells were transfected with reporter constructs containing either the wild-type (WT) *RBL1* promoter or the *RBL1* promoter with mutations in both E2F binding sites (E2F Double). Cells were serum-starved for 62 h, then either immediately harvested (0 h) or restimulated with serum for 24 h (24 h). Relative light units (RLUs) were calculated as the ratio of firefly luciferase activity from the promoter reporter constructs to the Renilla luciferase activity from a co-transfected control plasmid. One representative experiment from three biological replicates is shown. (**c**) Relative fold change in *RBL1* promoter activity after 24 h serum restimulation, compiled from three independent experiments represented in panel b. Bars represent fold change mean ± SEM. * Statistical significance between WT and E2F Double was calculated using an unpaired two-tailed Student’s *t*-test. (**d**). FACS analysis from cell samples used in panel (**b**,**c**), confirming the cell cycle distribution (G_0_ vs. 24 h).

As expected, *CCNE2* expression surged around 16 h post-stimulation, marking end of G1 and entry into S phase. On the other hand, *RBL1* levels peaked a bit later—around 20 h—while *CDC25C* followed at 24 h (Figure 3a). This temporal expression pattern suggested that *RBL1* peaks during S phase of the cell cycle.

To be able to assess the functions of the E2F binding sites in the *RBL1* promoter, we constructed a series of *RBL1* promoter luciferase reporter plasmids that contain either the wild-type *RBL1* promoter (WT) or different mutations for the E2F sites, such as proximal E2F site mutant (E2F Prox), distal E2F site mutant (E2F Dist), and a double mutant (E2F Double), in a fashion that any change in promoter expression will be reflected in a measurable luciferase signal (Table 1). Alignments of promoter sequences can be accessed in Supplementary Figure S3.

We began our luciferase reporter promoter analysis by assessing the cell cycle-dependent transcriptional regulation of *RBL1*. The wild-type (WT) promoter construct was compared against an E2F double mutant construct in NIH3T3 cells under G_0_ and serum-stimulated conditions. As expected, the WT promoter exhibited low activity in G_0_ and strong induction upon serum stimulation. In contrast, the E2F double mutant displayed elevated basal activity in G_0_, reflecting the loss of repression normally mediated by the tandem E2F sites during quiescence. Upon serum stimulation, the mutant promoter activity did not further increase, while the WT promoter was strongly induced, resulting in similar absolute luciferase levels between WT and mutant at 24 h (Figure 3b,c). FACS analysis confirmed proper cell cycle transitions (Figure 3d). These results indicate that the tandem E2F sites in the *RBL1* promoter mediate both repression during G_0_/G_1_ and cell cycle-dependent activation, consistent with previous findings [20].

### 2.4. p53-Dependent Repression of RBL1 Requires Both Tandem E2F Binding Sites

Previous studies demonstrated that the tandem E2F sites in the *RBL1* promoter are critical for its proper cell cycle regulation [20]. In addition, *RBL1* has been recognized as a target of the p53–p21–RB/DREAM pathways [16,18,37,38]. To confirm and extend these findings in the context of p53-mediated repression, we examined whether these conserved E2F motifs also mediate the p53-dependent downregulation of *RBL1*. We performed luciferase reporter assays in HCT116 cells co-transfected with either wild-type or mutant p53, along with a series of *RBL1* promoter constructs: wild-type (WT), proximal E2F site mutant (E2F Prox), distal E2F site mutant (E2F Dist), and a double mutant (E2F Double).

**Figure 4:**
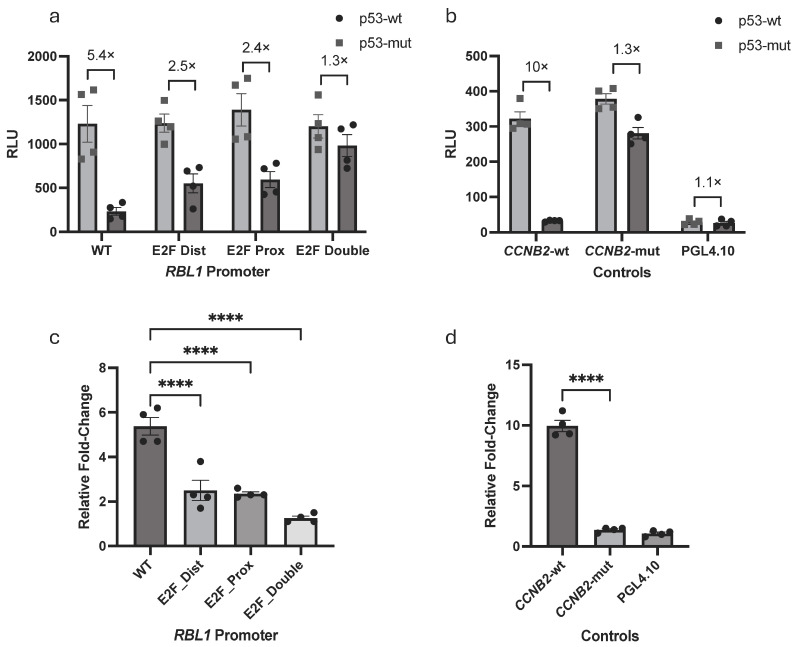
p53-Dependent Repression of *RBL1* Requires Both Tandem E2F Binding Sites (**a**) Luciferase reporter assay in HCT116 p53^−/−^ cells demonstrating the role of E2F binding sites in p53-dependent repression of *RBL1*. Cells were co-transfected with either a wild-type p53 (p53-wt) or a mutant p53-expressing plasmid (p53-mut), along with *RBL1* promoter constructs: wild-type (WT), E2F distal mutant (E2F Dist), E2F proximal mutant (E2F Prox), or E2F Double mutant (E2F Double). Relative light units (RLUs) were calculated as the ratio of firefly luciferase activity from the promoter reporter constructs to the Renilla luciferase activity from a co-transfected control plasmid. (**b**) control constructs for the Luciferase reporter assay shown in (**a**). *CCNB2* (Cyclin B2) promoter constructs (WT and mutant) was used as a positive control for p53-mediated repression, and an empty reporter vector (PGL4.10) as a negative control. Relative light units (RLUs) were calculated similarly to panel A. Mean values ± SEM from *n* > 3 biological replicates are shown. (**c**,**d**) shows the relative fold change in RBL1 promoter activity comparing p53-wt vs. p53-mut groups, compiled from independent experiments represented in panel (**a**,**b**). Bars represent fold change mean ± SEM. Statistical significance between WT and mutant promoters was calculated using Ordinary one-way ANOVA (****: *p* ≤ 0.0001).

Our results showed a gradient of repression: the wild-type promoter was strongly repressed by p53, while constructs with a single E2F mutation showed partial derepression. The double mutant, however, was completely resistant to p53 (Figure 4a). This possibly indicates that both E2F motifs contribute to repression and are likely to function in a cooperative or redundant manner.

This assay system was also validated by addition of *CCNB2* (Cyclin B2) promoter constructs (wild-type and mutant) or an empty luciferase vector which served as a negative control. The controls behaved exactly as anticipated (Figure 4b), which strengthened our confidence in the experimental outcome. Overall, these results confirm and extend prior work by showing that the tandem E2F sites are not only essential for cell cycle regulation of *RBL1*, but also represent the key elements through which p53 exerts transcriptional repression.

### 2.5. E2F1–4, RB, and p107 Bind to the RBL1 Promoter Through the Two E2F Binding Sites

To validate that these regulatory effects were due to direct binding at the *RBL1* promoter, we used both chromatin immunoprecipitation (ChIP) and in vitro DNA pull-down assays. ChIP was conducted in T98G cells. We examined binding of E2F1–4, p130, LIN37, LIN9, BMYB, FOXM1, and NFYA to the *RBL1* promoter across synchronized cells.

**Figure 5:**
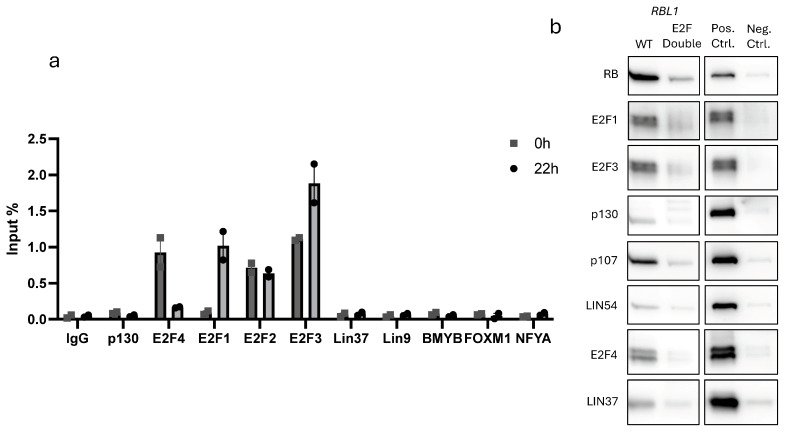
E2F1–4, RB, and p107 Bind to the *RBL1* Promoter through the two E2F Binding Sites. (**a**) In vivo protein binding to the *RBL1* promoter. Chromatin immunoprecipitation (ChIP) followed by real-time qPCR was performed in T98G cells synchronized to G0 (quiescent) or a later cell cycle phase (G2/M), to assess the binding of p130, E2F1, E2F2, E2F3, E2F4, LIN37, LIN9, BMYB, FOXM1, and NFYA. Mean values ± SEM are given of one representative experiment from three biological replicates. (**b**) In vitro protein binding to the *RBL1* promoter. Nuclear extracts from proliferating HCT116 cells were subjected to DNA affinity purification, employing biotinylated wild-type (WT) and E2F double mutant (E2F Double) *RBL1* promoter fragments. Binding of E2F1, E2F2, E2F3, E2F4, RB, p107, LIN54, LIN37, and p130 was analyzed by Western blot. Input samples were taken from the nuclear extract before purification. Cyclin B2 biotinylated wild-type probe was served as a positive control and GAPDH biotinylated wild-type probe served as a negative control. One representative experiment from *n* ≥ 2 biological replicates is shown. Complete presentation of the bolt and others from different biological replicates are shown in Supplementary Figure S4.

The results were as predicted. Activating E2Fs (E2F1–3) showed strong binding during cell cycle arrest as well as in cell cycle progression, with the exception of E2F1 that showed minimal binding in G_0_ but increased occupancy during cell cycle re-entry (Figure 5a). E2F4, known for its role in DREAM-mediated repression, bounds strongly in G_0_ [15,16].

For in vitro binding, we synthesized biotin-labeled *RBL1* promoter fragments and incubated them with nuclear extracts from proliferating HCT116 cells. The wild-type probe pulled down E2F1–4, as well as RB and p107, confirming their direct interaction. When we used a probe containing mutations in both E2F sites (E2F Double), this binding was dramatically reduced (Figure 5b). Notably, we also detected faint binding of DREAM components such as LIN54, LIN37, and p130. Although weaker, their presence suggests a minor yet potentially meaningful involvement in promoter repression. Collectively, our results confirm earlier functional studies showing that RB and p107 repress *RBL1* through E2F-binding sites and that E2F1–4, RB, and p107 bind the *RBL1* promoter [23,41]. Importantly, we confirm and extend these observations by providing direct biochemical evidence of this binding. Together, our results firmly establish the tandem E2F motifs as critical regulatory elements that mediate the context-dependent control of *RBL1* by RB family proteins and E2Fs.

### 2.6. p53-Dependent Repression of RBL1 Is Mediated by RB with Partial Support from DREAM

Finally, we sought to dissect the specific contributions of RB and DREAM to the repression of *RBL1* by p53. Using luciferase assays, we examined promoter activity in four HCT116 cell-lines: wild-type (WT), RB knockouts (RB^−/−^), LIN37 knockouts (LIN37^−/−^), and double knockouts lacking both RB and LIN37 (RB^−/−^; LIN37^−/−^). These cells were co-transfected with either wild-type or mutant p53 along with the *RBL1* WT promoter construct.

**Figure 6:**
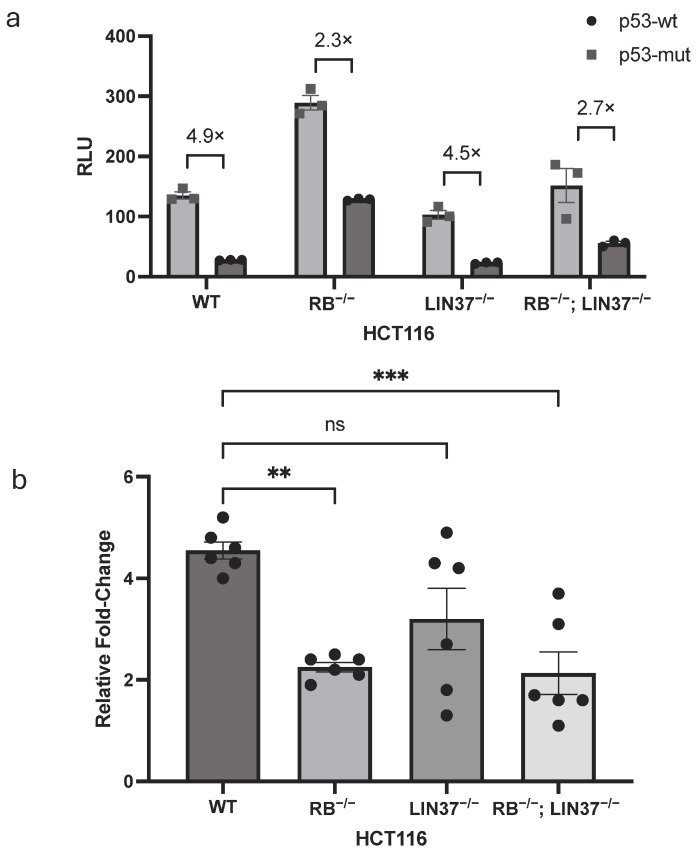
p53-dependent Repression of *RBL1* is mediated by RB with Partial Support from DREAM. (**a**) Luciferase reporter assay demonstrating the contribution of RB and DREAM to p53-dependent repression of *RBL1*. HCT116 wild-type (WT), RB-deficient (RB^−/−^), LIN37-deficient (LIN37^−/−^), and double knockout (DKO) cells (lacking both RB and LIN37) were co-transfected with either a wild-type p53 (p53-wt) or a mutant p53-expressing plasmid (p53-mut) along the wild-type *RBL1* promoter construct. Relative light units (RLUs) were calculated as the ratio of firefly luciferase activity from the promoter reporter constructs to the Renilla luciferase activity from a co-transfected control plasmid. Mean values ± SEM are from *n* > 3 replicates. (**b**) Compilation of relative fold changes in luciferase activity from *n* > 3 biological replicates represented in panel a. Bars show fold change mean ± SEM. Statistical significance between WT and knockouts was calculated using Ordinary one-way ANOVA (ns: not significant; **: *p* ≤ 0.01; ***: *p* ≤ 0.001).

In wild-type cells, p53 repressed promoter activity as expected. However, in cells lacking RB, this repression was massively weakened, suggesting that RB is the dominant effector in this pathway (Figure 6a). LIN37^−/−^ cells displayed only a modest loss of repression, indicating that DREAM contributes to a lesser extent. Interestingly, the RB;LIN37 double knockout cells did not show greater derepression than the RB single knockout, reinforcing the conclusion that RB is the primary mediator of the p53-dependent *RBL1* repression, and that the contribution of the DREAM complex—at least via LIN37—is minimal and non-additive. This pattern was consistent across biological replicates and was reflected in fold-change comparisons (Figure 6b). Together, these findings suggest a model where RB is the principal mediator of p53-induced repression at the *RBL1* promoter, while DREAM may enhance repression under certain conditions or in specific phases of the cell cycle.

## 3. Discussion

The tight regulation of cell cycle progression is essential for preserving cellular homeostasis, with the retinoblastoma (RB) tumor suppressor family and the p53 pathway playing central, interconnected roles [18]. Previous studies had already suggested that *RBL1* (p107) is repressed downstream of p53 via the p53–p21–RB/DREAM axis [35,38]. In this study, we systematically confirmed these findings and extended them by demonstrating that repression of *RBL1* is mediated specifically through two highly conserved tandem E2F binding sites in its promoter. Both sites proved indispensable for p53-dependent repression, underscoring their critical regulatory role. Furthermore, our work consolidates earlier observations that RB is the primary effector of this repression, while the DREAM complex makes only a supportive contribution. The importance of these components is underscored by our observation that LIN37 knockout cells exhibit only partial derepression of *RBL1*, whereas cells lacking both RB and LIN37 do not show further derepression beyond what is observed in RB-deficient cells, thereby reinforcing the conclusion that RB is essential, whereas DREAM is supportive but not sufficient in this context.

Initial validation of this regulatory mechanism came from our experiments in HCT116 cells, which confirmed earlier suggestions that *RBL1* is a p53-repressed gene and that repression is strictly p21-dependent [18,35,38]. Treatment with Nutlin-3a, a well-established activator of p53, resulted in a pronounced reduction in *RBL1* mRNA levels in wild-type HCT116 cells, whereas no repression was observed in p21-null (p21^−/−^) cells (Figure 1). These results consolidate prior knowledge by providing direct evidence in a human colorectal carcinoma model and firmly place *RBL1* among the class of genes indirectly repressed by p53 through the p53–p21–RB axis. Mechanistically, p21 inhibits CDK activity, leading to hypophosphorylation of RB-family proteins, which then engage with E2F transcription factors to assemble repressive complexes at E2F target promoters. Our findings thus confirm and extend genome-wide observations [37,38] that position p21 as the indispensable mediator that links p53 activation to the transcriptional repression of *RBL1*, in full agreement with the current mechanistic framework describing indirect E2F-target gene repression by p53 [18,42].

To refine our understanding of how this repression is executed, we compared a 2.9 kb-long promoter fragment to a 400 bp fragment and found that the minimal 400 bp fragment of the human *RBL1* promoter retained full p53-responsiveness, showing re-pression levels equivalent to those observed with the full-length 2.9 kb promoter (Figure 2a). This observation allowed us to pinpoint the key regulatory elements within a compact, evolutionarily conserved region. In line with this, our conservation analysis revealed that the two tandem E2F binding sites within this 400 bp region are remarkably conserved across species (Figure 2b), emphasizing their likely functional significance. These sites have been previously described and are known to mediate transcriptional repression by both RB and p107 proteins [23]. Furthermore, in addition to repression, these elements have also been shown to be required for cell cycle-regulated activation of the promoter, establishing their dual functional role [20]. Expanding on this foundation, our mutagenesis studies revealed that while individual mutation of either E2F site led to partial loss of p53-mediated repression, complete abrogation of repression occurred only when both sites were simultaneously mutated (Figure 4). These findings reinforce the established role of the tandem E2F motifs and extend it by showing that both sites act cooperatively to transmit p53–p21-dependent repression at the *RBL1* promoter.

**Figure 7:**
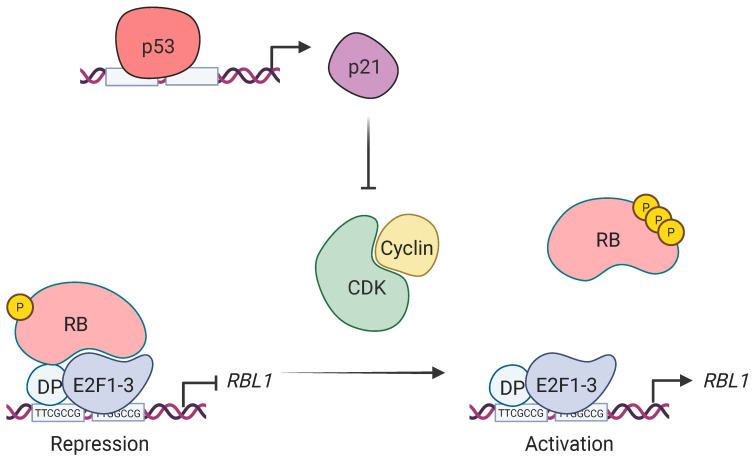
Proposed schematic model of p53-dependent transcriptional repression of *RBL1*. Upon activation, p53 transactivates *CDKN1A* (p21) by directly binding its promoter. The resulting p21 protein inhibits Cyclin-CDK complexes, preventing them from hyperphosphorylating RB pocket protein. In its hypophosphorylated state, RB form repressive complexes with E2F transcription factors and assemble at the tandem E2F sites in the *RBL1* promoter, repressing its transcription. In the absence of p53-p21 activation, active Cyclin-CDK hyperphosphorylates RB, disrupting repressor complexes and allowing activating E2Fs (E2F1–3) to bind the promoter throw the tandem E2F sites and induce *RBL1* transcription. Thus, p21 maintains *RBL1* repression by preserving the hypophosphorylated, repressive state of RB–E2F complexes. This image was created using Biorender.com (FT28O9ZM5P: Dated 25 August 2025).

To validate these findings, we complemented our promoter-reporter assays with direct binding analyses. Our chromatin immunoprecipitation (ChIP) experiments con-firmed the dynamic, cell cycle-dependent binding of E2F1, E2F2, E2F3, and E2F4 to the *RBL1* promoter in vivo, consistent with earlier reports that members of the RB family and E2Fs associate with this promoter [23,41] (Figure 5a). To further validate the specificity of these interactions, we performed in vitro DNA affinity purification using biotinylated promoter probes and found that E2F1, E2F3, E2F4, RB, and p107 all showed strong, specific binding to the wild-type promoter. Importantly, binding was either markedly reduced or entirely lost when both E2F sites were mutated in the Double E2F probe (Figure 5b), confirming that these motifs are the primary docking sites for repressor complex assembly. Although we also detected binding of MuvB components, including LIN54 and LIN37, their overall binding intensity was lower and were not consistent with ChIP assays. Due to technical issues with the RB antibody, we were unable to perform ChIP experiments in this study. However, our DNA affinity purification experiments demonstrate that RB specifically occupies the two tandem E2F sites in the *RBL1* promoter. While previous studies have shown RB binding to the *RBL1* promoter in the same cell type [41], our results specify that this interaction occurs directly at the tandem E2F sites. Together with our knockout data, these results confirm earlier functional observations and extend them by providing direct biochemical evidence that the tandem E2F motifs are the central regulatory elements through which RB family proteins and E2Fs control the p53-dependent transcriptional repression at the *RBL1* promoter, while E2F4 along with p107 and p130 might together induce minimal repression (Figure 7). Whether p107/p130-E2F4 repressing complex is bound to MuvB to make the DREAM complex at the *RBL1* promoter deserves further investigation in future studies.

The strong loss of repression in RB-knockout cells, compared to the relatively mild derepression observed in LIN37-deficient cells, highlights the essential role of RB in this context (Figure 6). This is in accordance with earlier studies demonstrating that p107 mRNA levels are elevated in RB^−/−^ fibroblasts but not in cells lacking p130, especially under serum-starved conditions [35]. Moreover, elevated *RBL1* expression has been reported across various RB-deficient models, suggesting that RB plays a non-redundant role in *RBL1* transcriptional regulation [36]. Our findings build upon this model by showing that *RBL1* is repressed by p53 in a p21-dependent manner, hyper-phosphorylating the RB pocket protein and causing it to assemble repressing complexes with E2F1-3 at the tandem E2F sites, hence, downregulating the *RBL1* promoter (Figure 7).

The implications of this mechanism go beyond transcriptional regulation. Our findings align with the broader biological role of p107 as a compensatory tumor suppressor, particularly in the context of RB loss. Genetic studies have shown that RB/p107 and RB/p130 double-knockout mice develop retinoblastoma, while single knockouts do not [24,25,26]. This redundancy appears to extend to other tissues, as similar compensation has been observed in heart, lung, and skin tissues [31,32,33]. It is also well-documented that p107 levels increase following RB loss, both at the transcriptional and post-transcriptional levels, supporting the idea of a feedback compensation mechanism rather than simple redundancy [28,34,35]. Our results confirm this framework and extend it by providing a molecular basis for this feedback, where *RBL1* is not only a functional compensator for RB loss, but also directly regulated by the same network that enforces cell cycle arrest via p53 and p21, thus integrating it into the broader tumor suppressor circuitry. A report in 2019 showed that p107 can repress G2/M genes and regulate mitotic entry in the absence of RB and p130, despite being normally expressed during S phase [43]. This suggests that under specific conditions—such as a loss of a pocket protein or p53 activation—p107 expression can extend beyond its canonical timing and exert repressive effects through alternative complexes, potentially involving E2F4–MuvB. This functional plasticity is further underscored by mouse knockout models, where deletion of both p107 and p130 leads to severe developmental defects, while single knockouts are largely tolerated [33,44,45,46]. Collectively, these results emphasize the flexibility of the RB family in maintaining transcriptional repression and point to the need for precise control of *RBL1* expression during stress or transformation.

Interestingly, the p53-dependent repression of *RBL1* may at first seem paradoxical, particularly given that p107 is generally associated with growth arrest and senescence. One might anticipate that p53 would upregulate *RBL1* to enforce arrest. However, previous studies and our present data suggest a more nuanced, context-dependent role for p107. In scenarios where RB and p130 are absent, p107 might act outside its usual window to compensate by repressing S or G2/M phase genes. Therefore, p53-mediated repression of *RBL1* may help limit p107’s compensatory activity under certain stress contexts, preventing inappropriate activation of repressive programs or tuning the cellular response. This reflects a delicately balanced transcriptional network, where selective repression complements transcriptional activation to fulfill the tumor suppressor function of p53.

These insights carry important implications for cancer biology and therapy. By confirming *RBL1* as a p53-repressed gene that requires both tandem E2F sites and the p21–RB pathway, our work underscores the importance of this regulatory axis in tumor suppression. In tumors with functional p53 but loss of RB, compensatory upregulation of *RBL1* could serve as a secondary brake on cell proliferation. While our use of LIN37^−/−^ cells helped evaluate the role of DREAM, the strong binding of E2F4 to the *RBL1* promoter raises further questions. E2F4 may function independently or in association with p130, not necessarily as a part of the DREAM complex [13,47]. Schade et al. (2019) reported that knockdown of p130 caused slight increase in p107 levels, implicating the contribution of p130—possibly by associating with E2F4— in repressing *RBL1* expression [43]. This suggests that disrupting p130–E2F4-mediated repression might be a viable therapeutic approach to restore or enhance *RBL1* expression in RB-deficient cancers. Further studies will be needed to clarify the distinct contributions of E2F4 and p130 to determine this alternative repression axis, which could reveal actionable nodes to reinforce tumor suppressive programs in RB-compromised settings.

In conclusion, our study confirms that the p53–p21 pathway represses *RBL1* expression through a mechanism requiring tandem E2F binding sites, with RB serving as the dominant mediator and DREAM contributing in a more limited capacity. These results consolidate and extend earlier findings by providing direct biochemical evidence of factor binding at the *RBL1* promoter. By placing *RBL1* at the intersection of p53-mediated repression, RB-family feedback, and compensatory tumor suppressor functions, this work refines our understanding of how transcriptional control of *RBL1* integrates into broader cell cycle regulation. Such insights provide a framework for future studies aimed at disentangling the relative contributions of RB, p107, p130, and E2F4 in normal physiology and in cancer, where this regulatory circuitry may be disrupted.

## 4. Materials and Methods

### 4.1. Cell Lines and Knockouts

Parental human colon carcinoma HCT116 cells and HCT116 cells with targeted deletions in both p53 (HCT116 p53^−/−^) or p21WAF1/CIP1 (HCT116 p21^−/−^) alleles were kindly provided by Bert Vogelstein (Johns Hopkins University, Baltimore, MD, USA) [48]. Additional HCT116 knockout cell lines (RB^−/−^, LIN37^−/−^, and the double knockout RB^−/−^; LIN37^−/−^) were generated using CRISPR/Cas9 nickase-mediated genome editing as previously described [49,50] and were kindly provided by Kurt Engeland (University of Leipzig, Leipzig, Germany), along with NIH3T3 fibroblasts.

### 4.2. Cell Culture and Treatments

All cell lines were cultured in Dulbecco’s modified Eagle’s medium (DMEM; Gibco, Grand Island, NY, USA) supplemented with 10% fetal calf serum (FCS; Biochrom, Cambridge, UK) and 1% penicillin-streptomycin (Sigma-Aldrich, St. Louis, MO, USA) at 37 °C in a humidified incubator with 10% CO_2_. For p53 activation, HCT116 cells were treated with 10 µM Nutlin-3a (Cayman Chemicals, Ann Arbor, MI, USA) or DMSO as control for 48 or 72 h. For cell cycle synchronization, NIH3T3 cells were serum-starved in 0% FCS DMEM for 62 h to induce G_0_ arrest, followed by stimulation with 20% FCS DMEM to induce synchronous re-entry into the cell cycle.

### 4.3. Flow Cytometry

For cell cycle analysis, cells were fixed in 75% ethanol at −20 °C for at least 24 h. Fixed cells were stained with 100 µg/mL propidium iodide in PBS supplemented with 1 mM EDTA and 1:1000 RNaseA and analyzed on an LSR II flow cytometer (BD Biosciences, San Jose, CA, USA). Data were analyzed using FlowJo software, version 10.8.1 (BD Biosciences).

### 4.4. RNA Isolation and Quantitative RT-PCR

Total RNA was extracted using TRIzol reagent (ThermoFisher Scientific, Waltham, MA, USA) according to the manufacturer’s instructions. Semi-quantitative real-time PCR was performed using the GoTaq^®^ 1-Step RT-qPCR System (Promega, Madison, WI, USA) on an ABI 7300 Real-Time PCR System (Applied Biosystems, Foster City, CA, USA) with SYBR Green detection. Relative expression levels were calculated by the ΔΔCt method. Primer sequences are listed in Supplementary Table S1.

### 4.5. Cloning and Mutagenesis

The *RBL1* promoters (Long and Short) were PCR-amplified from human genomic DNA of HCT116 cells and cloned into the multiple cloning site of the pGL4.10[luc2] vector (Promega, Madison, WI, USA), upstream of the firefly luciferase reporter gene. Primer sequences used for cloning are listed in Supplementary Table S1. Mutations in the two tandem E2F binding sites within the *RBL1* promoter were introduced using the QuikChange II site-directed mutagenesis kit (Agilent Technologies, Santa Clara, CA, USA) according to the manufacturer’s protocol. Mutagenic primers were designed to introduce specific base substitutions that disrupt E2F consensus motifs without altering adjacent sequences (see Supplementary Table S1). PCR reactions were performed using high-fidelity DNA polymerase provided with the kit, followed by DpnI digestion to remove methylated parental plasmid DNA. The resulting constructs—wild-type and E2F-mutant versions of both the long and short *RBL1* promoters—were verified by Sanger sequencing prior to use in luciferase reporter assays and DNA affinity purification experiments.

The human p53 expression plasmids pcDNA-p53wt and pcDNA-p53mut were produced by amplifying the insert of pCMV-p53wt and pCMV-p53mut R175H (kindly provided by Bert Vogelstein) and ligating into pcDNA3.1HisC (Invitrogen, Carlsbad, CA, USA) [6]. The p53-R175H loses the transactivating function of the wild-type p53 due to its defective DNA binding interface [51].

### 4.6. Luciferase Promoter Reporter Assays

HCT116 or NIH3T3 cells were seeded in 12- or 24-well plates (50k/well) and transfected with 250 ng luciferase reporter constructs using GeneJuice (Millipore, Darmstadt, Germany) or PEI (polyethylenimine). For normalization, 25 ng of Renilla luciferase vector (pGL4.70[hRluc], Promega, Madison, WI, USA) was co-transfected. In p53-response assays 25 ng of p53-wt or p53-mut expression plasmids were co-transfected. Twenty-four hours after transfection, cells were lysed by being washed once with phosphate-buffered saline then lysis buffer was added. Luciferase activity was measured using the Dual-Luciferase Reporter Assay System (Promega, Madison, WI, USA) on a Glomax luminometer (Promega, Madison, WI, USA). Data were normalized as Firefly/Renilla ratios, and results are shown as relative luciferase units (RLUs).

### 4.7. Chromatin Immunoprecipitation (ChIP)

To investigate the in vivo binding of transcription factors to the *RBL1* promoter, we performed ChIP assays in synchronously growing T98G cells as described earlier [52,53,54,55]. Cells were crosslinked with 1% formaldehyde, lysed, and subjected to chromatin shearing by sonication to obtain DNA fragments averaging 200–500 bp. Immunoprecipitations were carried out overnight using respective antibodies (see Supplementary Table S2). A rabbit IgG antibody was used as a negative control. Following reverse crosslinking and DNA purification, qPCR was performed using primers spanning the conserved tandem E2F binding sites in the *RBL1* promoter. ChIP enrichment was quantified relative to input and IgG controls and normalized across experiments.

### 4.8. DNA Affinity Purification

To identify proteins that directly bind the *RBL1* promoter in vitro, we performed DNA affinity purification as previously described [56]. The assay was performed using biotinylated DNA probes containing either the wild-type or Double E2F-mutated *RBL1* promoter sequences. Biotinylated probes were generated by PCR amplification from pGL4.10-based *RBL1* luciferase reporter plasmids using specific primers (See Supplementary Table S1). Nuclear extracts were prepared from asynchronously growing HCT116 cells and incubated with streptavidin-coated magnetic beads pre-bound to the biotinylated DNA probes. After extensive washing, bound proteins were eluted, resolved by SDS-PAGE, and analyzed by Western blotting. The presence of specific transcriptional regulators—including E2F1, E2F3, E2F4, RB, p107, LIN37, LIN54, and p130—was assessed using antibodies validated for ChIP or immunoblotting (For antibodies details, check Supplementary Table S2). Cyclin B2 probe was included as a positive control, and a GAPDH proble was included as a negative control.

### 4.9. SDS-PAGE and Western Blotting

Protein samples (15–20 µg) were mixed with Laemmli buffer, denatured at 95 °C for 5 min, and separated on 10% SDS-polyacrylamide gels. Electrophoresis was performed in SDS running buffer using stepwise voltage increments (80 V to 180 V). Proteins were transferred to PVDF membranes using a semi-dry blotting system at 37 mA for 1.5 h. Membranes were blocked in 5% fetal calf serum (FCS) diluted in Tris-buffered saline with 0.1% Tween-20 (TBS-T) for 30–60 min at room temperature, then incubated overnight at 4 °C with primary antibodies diluted in 2.5% FCS/TBS-T. After washing, membranes were incubated with HRP-conjugated secondary antibodies for 1–2 h at room temperature. Chemiluminescent signals were developed using SuperSignal West Dura or Femto substrate (Thermo Fisher Scientific, Waltham, MA, USA) and visualized with a ChemoStar imaging system (Intas, Ahmedabad, India). Primary antibodies included E2F1, E2F3, E2F4, RB, p107, p130, LIN37 and LIN54 (see Supplementary Table S2 for antibody details).

### 4.10. Statistics

Data are presented as means ± standard error of the mean. The significance of the difference between two groups was assessed using unpaired two-tailed Student’s *t*-test or Ordinary one-way ANOVA. * *p* ≤ 0.05; ** *p* ≤ 0.01; *** *p* ≤ 0.001; **** *p* ≤ 0.0001; n.s.: not significant.

## Figures and Tables

**Table 1:** Mutations introduced into the tandem E2F binding sites of the human *RBL1* promoter.

Promoter		−27 E2F Dist	E2F Prox −9	TSS
Human *RBL1* WT	5′-CAGCTGCAGA	T**TTTCGCGC**G	C**TTTGGCGC**A	GGTGGTTGTG-3′
Human *RBL1* E2F Proximal	5′-CAGCTGCAGA	T**TTTCGCGC**G	C**AGCTCACC**A	GGTGGTTGTG-3′
Human *RBL1* E2F Distal	5′-CAGCTGCAGA	T**CACTCGAC**G	C**TTTGGCGC**A	GGTGGTTGTG-3′
Human *RBL1* E2F Double	5′-CAGCTGCAGA	T**CACTCGAC**G	C**AGCTCACC**A	GGTGGTTGTG-3′

## Data Availability

The original contributions presented in this study are included in the article/Supplementary Materials.

## Supplementary Materials

The supporting information can be downloaded at https://www.mdpi.com/article/10.3390/ijms26209903/s1.

## Abbreviations

The following abbreviations are used in this manuscript:

BMYB	B-Myb transcription factor
β-Actin	Beta-actin
CDK	Cyclin-dependent kinase
CDKN1A	Cyclin-dependent kinase inhibitor 1A (p21)
CDC25C	Cell division cycle 25C phosphatase
ChIP	Chromatin immunoprecipitation
DMSO	Dimethyl sulfoxide
DREAM	Dimerization partner, RB-like, E2F, and MuvB complex
E2F	E2 promoter-binding factor
FACS	Fluorescence-activated cell sorting
FCS	Fetal calf serum
FOXM1	Forkhead box M1 transcription factor
GAPDH	Glyceraldehyde-3-phosphate dehydrogenase
HRP	Horseradish peroxidase
LIN37	Lin-37 cell cycle regulatory protein
LIN54	Lin-54 cell cycle regulatory protein
LIN9	Lin-9 cell cycle regulatory protein
MDM2	Mouse double minute 2 homolog
mRNA	Messenger ribonucleic acid
MuvB	Multi-vulva class B core complex
NFYA	Nuclear transcription factor Y subunit alpha
PBS	Phosphate-buffered saline
PCR	Polymerase chain reaction
PVDF	Polyvinylidene difluoride
RB	Retinoblastoma protein
RBL1	Retinoblastoma-like protein 1 (p107)
RLU	Relative light unit
RT-qPCR	Reverse transcription quantitative polymerase chain reaction
SDS-PAGE	Sodium dodecyl sulfate polyacrylamide gel electrophoresis
SEM	Standard error of the mean
TBS-T	Tris-buffered saline with Tween 20
U6	U6 small nuclear RNA
WT	Wild-type
