# Supplementary material for: The Tumor Suppressor p53 Downregulates p107 (RBL1) Through p21–RB/E2F Signaling and Tandem E2F Sites
Source: Int J Mol Sci. 2025 Oct 11;26(20):9903. doi: 10.3390/ijms26209903 (PMC12564276; doi:10.3390/ijms26209903)
Supplement: Supplementary file 1 [file ijms-26-09903-s001.zip › Supplementary Figures.pdf]

Supplementary Figures

Suppl. Figure S1. Validation of p53 and p21 Knockout in HCT116 Cells

A. Western blot analysis confirming the knockout of p21 protein in HCT116 cell lines. Cell lysates from wild-type (WT), $p53^{-}/^{-}$, and $p21^{-}/^{-}$ HCT116 cells were probed with antibodies against p21. β-Actin was used as a loading control to ensure equal protein loading across all samples. **B.** Real-time RT-qPCR analysis of CDKN1A (p21) mRNA levels in WT and $p21^{-}/^{-}$ HCT116 cells treated with Nutlin-3a (10 µM) or DMSO (control) for 48 hours. CDKN1A expression was normalized to U6, and relative levels were calculated using the ΔΔCt method. Mean ± SEM from three biological replicates are shown.

Suppl. Figure S2. FACS analysis of NIH3T3 Cells.

Flow cytometry analysis (propidium iodide staining) of mouse NIH3T3 cells synchronized by serum starvation for 72 hours, followed by serum re-stimulation to induce synchronous re-entry into the cell cycle. Samples were collected every 4 hours post-stimulation and analyzed to determine DNA content and estimate cell cycle phase distribution over time.

Suppl. Figure S3. Sequence Alignment of Constructed *RBL1* Promoters

Alignment of the human RBL1 promoter (short fragment, ~400 bp) used in luciferase reporter assays, comparing the wild-type (WT) sequence to constructs carrying targeted mutations in the proximal E2F site (E2F Prox), distal E2F site (E2F Dist), or both sites (E2F Double). The wild-type reference sequence was obtained from the UCSC Genome Browser (human hg38). Mutated bases are highlighted, and the locations of the conserved E2F binding sites are indicated. These constructs were used in transcriptional assays described in Table 1 and Figure 4 to dissect the contribution of each E2F site to RBL1 promoter regulation.

Suppl. Figure S4. Full Western Blots for DNA Affinity Purification Assays

Complete Western blot image corresponding to the DNA affinity purification experiment. Antibodies against E2F1, E2F2, E2F3, E2F4, RB, p107, LIN54, LIN37, p130, and Histone 3 were used. Histone 3 was used as a loading control. Cyclin B2 was included as a positive control and GAPDH as a negative control. DHFR WT and Mut probes were included as an example of a gene that binds to most of these poteins diffirentially. Input samples (pre-purification nuclear extracts) are shown for comparison. Other biological replicates are also shown.

Suppl. Figure S1. Validation of p53 and p21 Knockout in HCT116 Cells

A

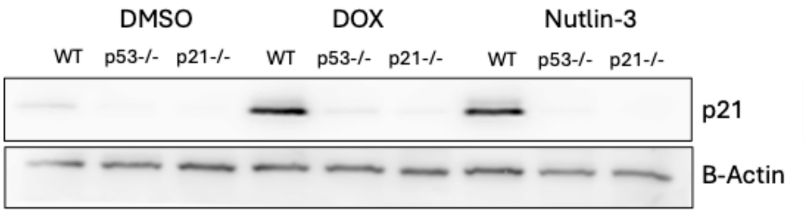

B

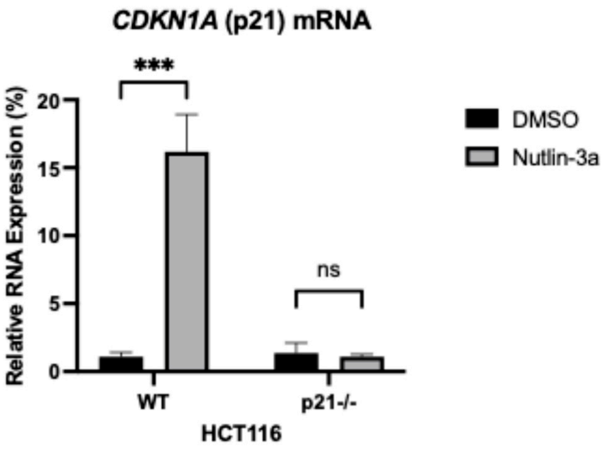

Suppl. Figure S2. FACS analysis of NIH3T3 Cells.

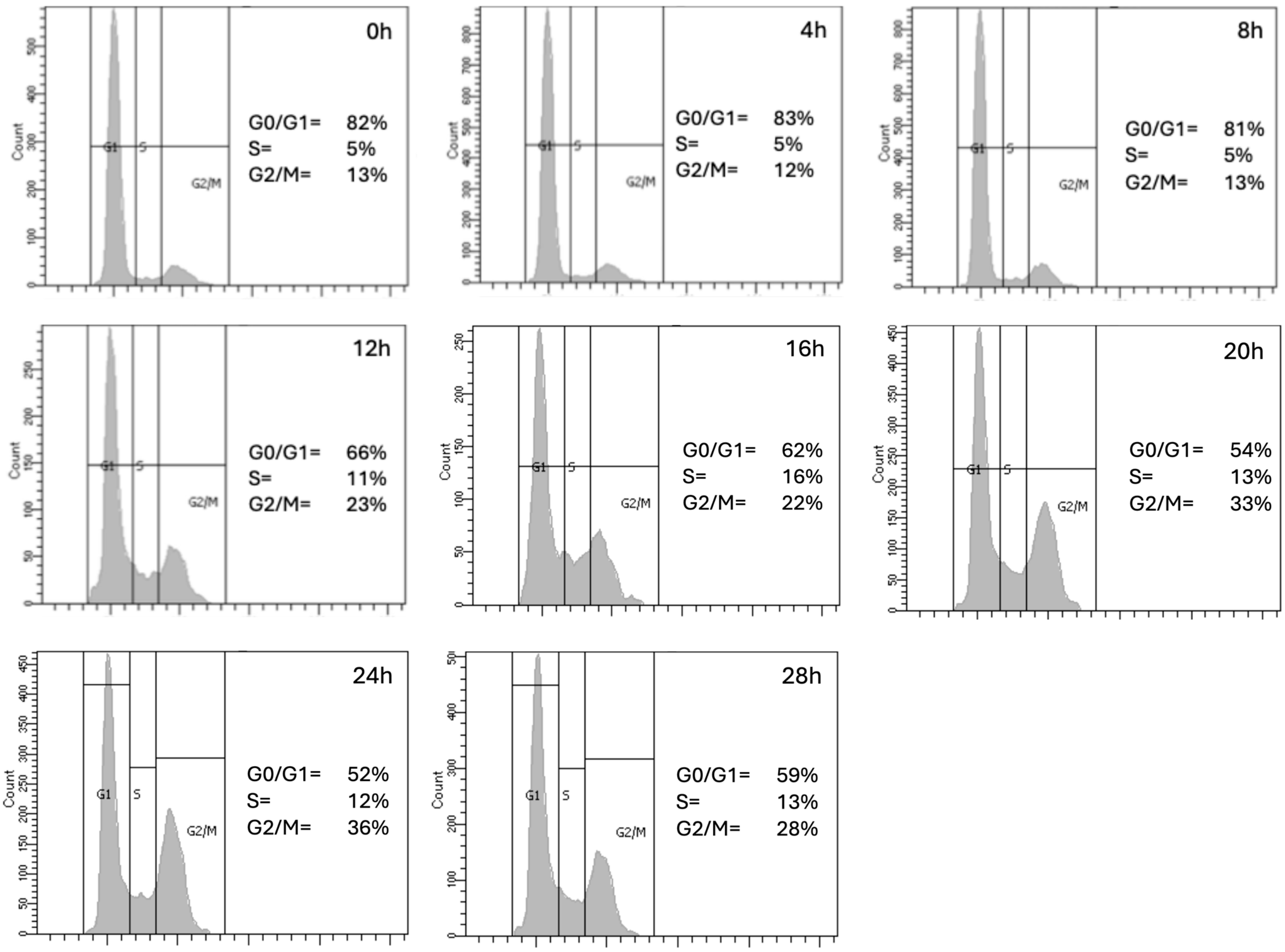

Suppl. Figure S3. Sequence Alignment of Constructed *RBL1* Promoters

	5	15	25	35	45	55
Reference (hg38)	ACCCAAGAGC	CTAACCAGGA	AGACAGGGGG	AGGCCGCGGG	CTTCATCTCC	CAAGAGATGG
WT (Short)	ACCCAAGAGC	CTAACCAGGA	AGACAGGGGG	AGGCCGCGGG	CTTCATCTCC	CAAGAGATGG
E2F Proximal	ACCCAAGAGC	CTAACCAGGA	AGACAGGGGG	AGGCCGCGGG	CTTCATCTCC	CAAGAGATGG
E2F Distal	ACCCAAGAGC	CTAACCAGGA	AGACAGGGGG	AGGCCGCGGG	CTTCATCTCC	CAAGAGATGG
E2F Double	ACCCAAGAGC	CTAACCAGGA	AGACAGGGGG	AGGCCGCGGG	CTTCATCTCC	CAAGAGATGG

	65	75	85	95	105	115
Reference (hg38)	ACTACACCTC	CCAGCAGGCT	CTGCGCGCGG	GCTGAGGATC	CCTCCGCTCT	TTTTCTGTCC
WT (Short)	ACTACACCTC	CCAGCAGGCT	CTGCGCGCGG	GCTGAGGATC	CCTCCGCTCT	TTTTCTGTCC
E2F Proximal	ACTACACCTC	CCAGCAGGCT	CTGCGCGCGG	GCTGAGGATC	CCTCCGCTCT	TTTTCTGTCC
E2F Distal	ACTACACCTC	CCAGCAGGCT	CTGCGCGCGG	GCTGAGGATC	CCTCCGCTCT	TTTTCTGTCC
E2F Double	ACTACACCTC	CCAGCAGGCT	CTGCGCGCGG	GCTGAGGATC	CCTCCGCTCT	TTTTCTGTCC

	125	135	145	155	165	175
Reference (hg38)	CGCCGGCTGG	GCCCCCCGCG	ACCAGCCAAG	GGCCAAGGAC	AGGTCTTTCA	GAATCTGAGG
WT (Short)	CGCCGGCTGG	GCCCCCCGCG	ACCAGCCAAG	GGCCAAGGAC	AGGTCTTTCA	GAATCTGAGG
E2F Proximal	CGCCGGCTGG	GCCCCCCGCG	ACCAGCCAAG	GGCCAAGGAC	AGGTCTTTCA	GAATCTGAGG
E2F Distal	CGCCGGCTGG	GCCCCCCGCG	ACCAGCCAAG	GGCCAAGGAC	AGGTCTTTCA	GAATCTGAGG
E2F Double	CGCCGGCTGG	GCCCCCCGCG	ACCAGCCAAG	GGCCAAGGAC	AGGTCTTTCA	GAATCTGAGG

	185	195	205	215	225	235
Reference (hg38)	TACATCTTCT	TATCACATTT	CCGGGGAGGG	ACTGCTAGGA	GCTCCGGAGG	AAAAACGGAC
WT (Short)	TACATCTTCT	TATCACATTT	CCGGGGAGGG	ACTGCTAGGA	GCTCCGGAGG	AAAAACGGAC
E2F Proximal	TACATCTTCT	TATCACATTT	CCGGGGAGGG	ACTGCTAGGA	GCTCCGGAGG	AAAAACGGAC
E2F Distal	TACATCTTCT	TATCACATTT	CCGGGGAGGG	ACTGCTAGGA	GCTCCGGAGG	AAAAACGGAC
E2F Double	TACATCTTCT	TATCACATTT	CCGGGGAGGG	ACTGCTAGGA	GCTCCGGAGG	AAAAACGGAC

	245	255	265	275	285	295
Reference (hg38)	TTTTTTTGAG	GAGAAAAGCG	GAGGCAGACG	GTGGATGACA	ACACGTCCCG	CAGCTGCAGA
WT (Short)	TTTTTTTGAG	GAGAAAAGCG	GAGGCAGACG	GTGGATGACA	ACACGTCCCG	CAGCTGCAGA
E2F Proximal	TTTTTTTGAG	GAGAAAAGCG	GAGGCAGACG	GTGGATGACA	ACACGTCCCG	CAGCTGCAGA
E2F Distal	TTTTTTTGAG	GAGAAAAGCG	GAGGCAGACG	GTGGATGACA	ACACGTCCCG	CAGCTGCAGA
E2F Double	TTTTTTTGAG	GAGAAAAGCG	GAGGCAGACG	GTGGATGACA	ACACGTCCCG	CAGCTGCAGA

	305	315	325	335	345	355
Reference (hg38)	TTTTCGCGCG	CTTTGGCGCA	GGTGGTTGTG	GGTAGCGCGC	CTGGGAGGGA	GAAAGAAGTC
WT (Short)	T**TTTCGCGC**G	C**TTTGGCGC**A	GGTGGTTGTG	GGTAGCGCGC	CTGGGAGGGA	GAAAGAAGTC
E2F Proximal	TTTTCGCGCG	C**AGCTCAC**CA	GGTGGTTGTG	GGTAGCGCGC	CTGGGAGGGA	GAAAGAAGTC
E2F Distal	T**CACTCGA**CG	CTTTGGCGCA	GGTGGTTGTG	GGTAGCGCGC	CTGGGAGGGA	GAAAGAAGTC
E2F Double	T**CACTCGA**CG	C**AGCTCAC**CA	GGTGGTTGTG	GGTAGCGCGC	CTGGGAGGGA	GAAAGAAGTC

	365	375	385	
Reference (hg38)	GGGGGCCGTG	GCGCGCAGCC	CGCGGGGCCT	GAAG
WT (Short)	GGGGGCCGTG	GCGCGCAGCC	CGCGGGGCCT	GAAG
E2F Proximal	GGGGGCCGTG	GCGCGCAGCC	CGCGGGGCCT	GAAG
E2F Distal	GGGGGCCGTG	GCGCGCAGCC	CGCGGGGCCT	GAAG
E2F Double	GGGGGCCGTG	GCGCGCAGCC	CGCGGGGCCT	GAAG

Suppl. Figure S4. Full Blots for DNA Affinity Purification Assays

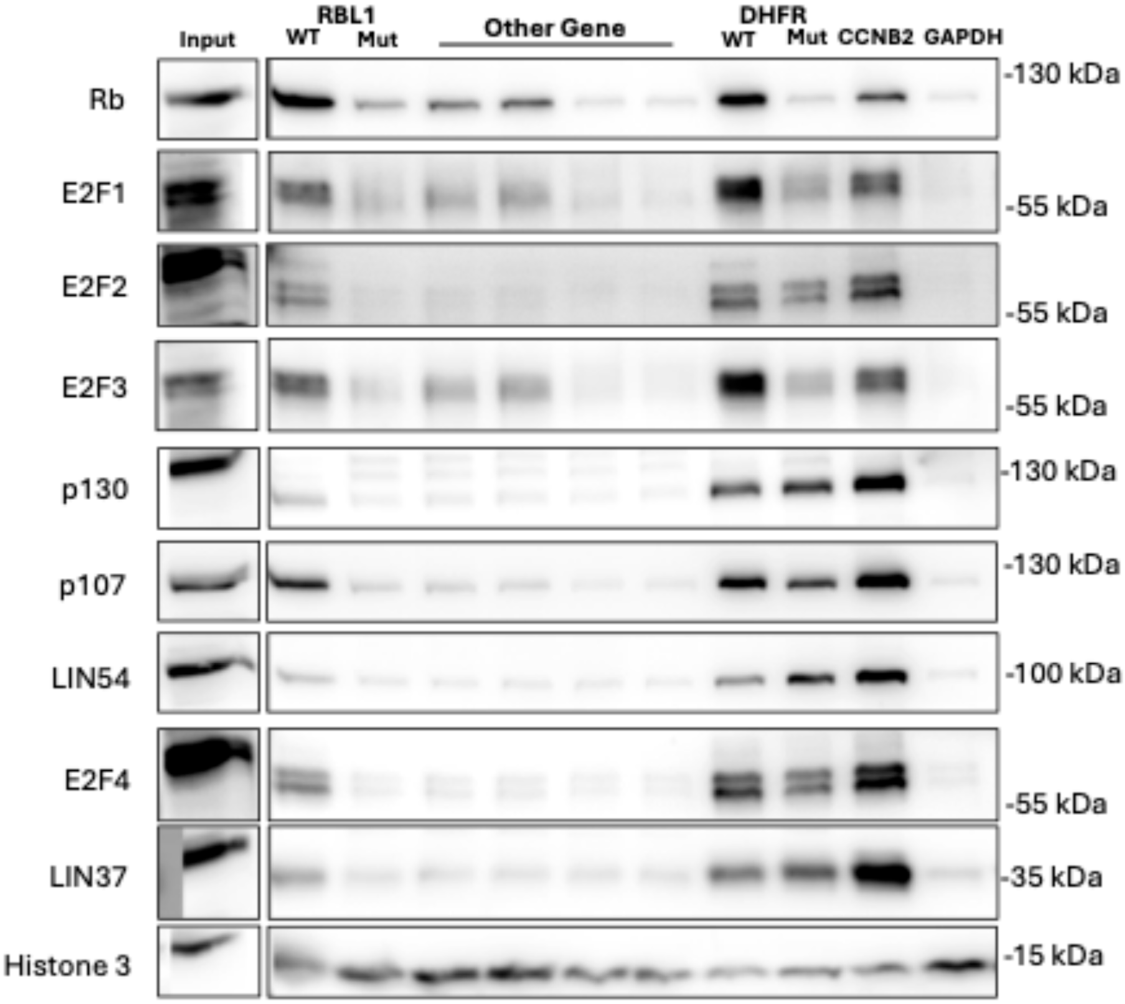

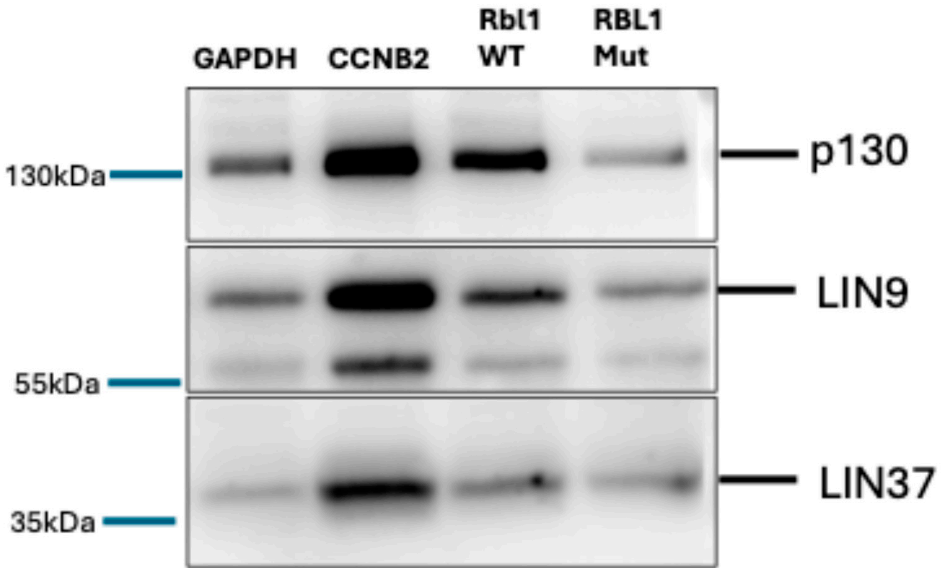
GAPDH
CCNB2
Rbl1 WT
RBL1 Mut
130kDa
55kDa
35kDa
p130
LIN9
LIN37

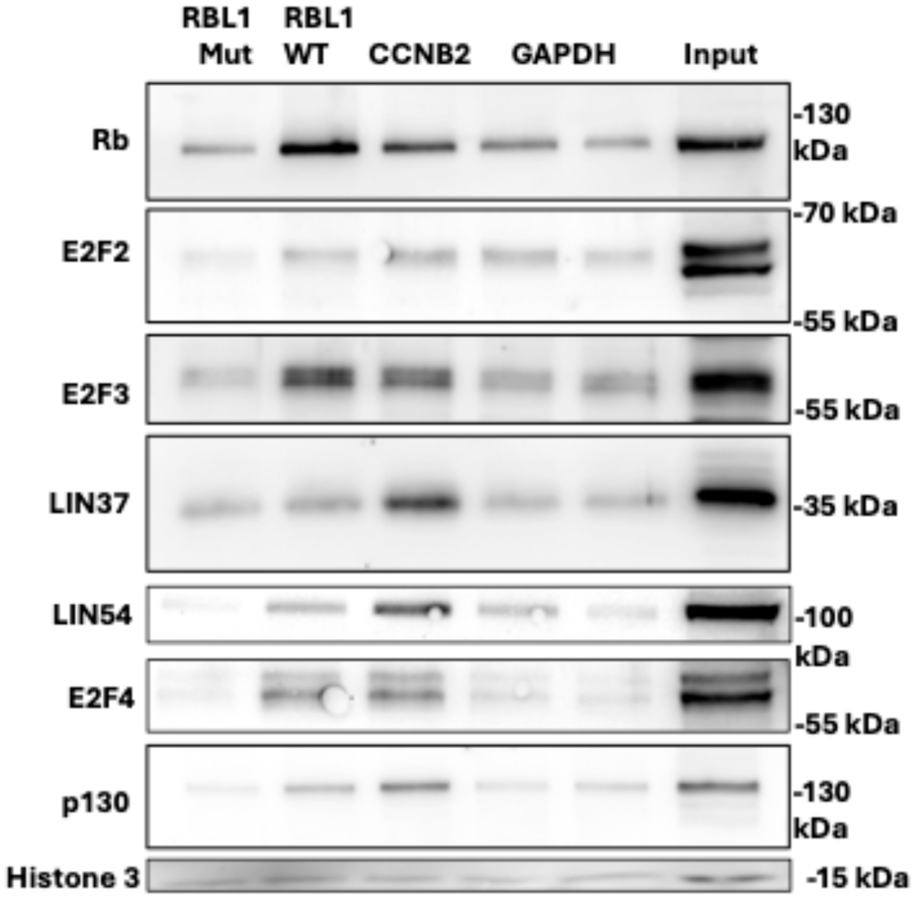
RBL1 Mut
RBL1 WT
CCNB2
GAPDH
Input
Rb
E2F2
E2F3
LIN37
LIN54
E2F4
p130
Histone 3
-130 kDa
-70 kDa
-55 kDa
-55 kDa
-35 kDa
-100 kDa
-55 kDa
-130 kDa
-15 kDa
